# Cutaneous leiomyoma in a child: A case report

**DOI:** 10.3892/ol.2013.1194

**Published:** 2013-02-15

**Authors:** NURSEL DILEK, DERYA YÜKSEL, İBRAHIM ŞEHITOĞLU, YUNUS SARAL

**Affiliations:** 1 Departments of Dermatology, Recep Tayyip Erdoğan University Medical Faculty Hospital, Rize 53000, Turkey; 2 Pathology, Recep Tayyip Erdoğan University Medical Faculty Hospital, Rize 53000, Turkey

**Keywords:** leiomyoma, child, cancer

## Abstract

Leiomyoma is a benign tumour commonly encountered in the genitourinary and gastrointestinal organs in adults. Cutaneous leiomyomas are rare benign tumors arising from the arrector pili muscle of hair follicles. Cutaneous leiomyomas are more likely to occur in adults than in children. We describe a case of a 10-year-old female who presented with multiple, firm, red-brown masses on the back. A punch biopsy was performed. Under high-power examination, spindle cells with an eosinophilic cytoplasm were observed and immunohistochemical studies were performed; the cells stained strongly positive for smooth muscle actin (SMA). The patient was subsequently diagnosed with pilar leiomyoma and referred to a plastic surgeon for surgical treatment. Although cutaneous leiomyoma is a rare disorder, we identified a case of pilar leiomyoma in a young female. A careful clinical assessment led to the correct diagnosis and therapy in the present case. We propose that leiomyoma ought to be considered in the differential diagnosis of any cutaneous or mucosal mass in children.

## Introduction

Leiomyoma is a benign tumour commonly encountered in the genitourinary and gastrointestinal organs in adults (1). Cutaneous leiomyomas are rare benign tumors arising from the arrector pili muscle of hair follicles, ranging in number from a few to several hundred (2,3). The skin is the second most common location for leiomyoma after the uterus, hosting ∼5% of all leiomyomas (4). According to their site of origin, leiomyomas may be classified into three types: i) piloleiomyomas, ii) angioleiomyomas and iii) dartoic leiomyomas. Piloleiomyomas are derived from the arrector pili muscle of hair follicles, whereas angioleiomyomas include those originating from the vascular smooth muscle and dartoic leiomyomas consist of those originating from the smooth muscle of genital skin (5). Cutaneous leiomyomas are more likely to occur in adults than in children, and often arise in the fifth and sixth decades of life (6). These lesions may be hereditary or sporadic (7).

## Case report

**Figure 1:**
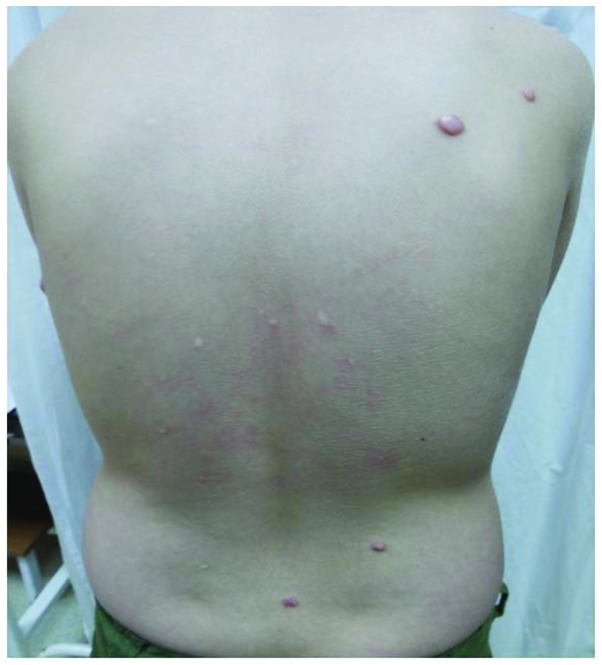
Multiple, firm, red-brown masses in the left scapular region.

**Figure 2:**
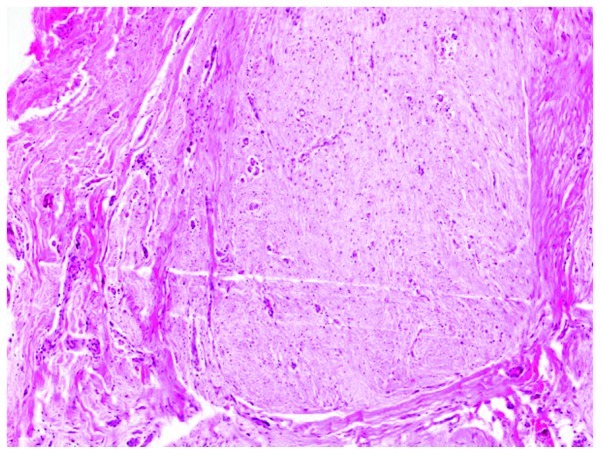
Spindle cells with an eosinophilic cytoplasm.

**Figure 3:**
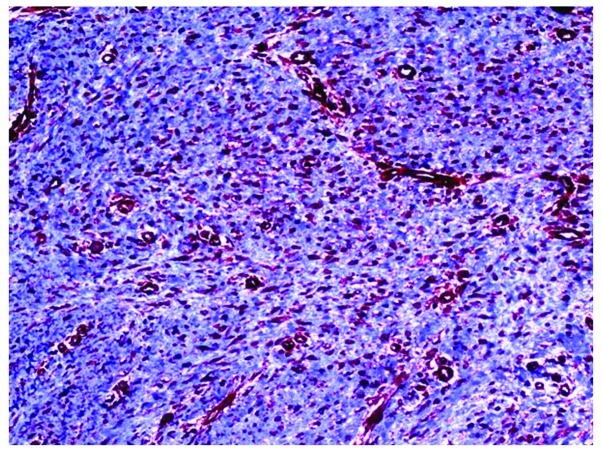
Immunohistochemical staining for smooth muscle actin (SMA).

In the present study, we describe a case of a 10-year-old female submitted to the Dermatology Department of Recep Tayyip Erdoğan University Medical Faculty, Rize, Turkey, with a two-month history of a lesion in the right scapular and lumbar regions. During the second month, the mass was observed to have increased in size and become painful. On clinical examination, multiple firm red-brown masses were observed on the back of the patient, the largest of which measured ∼10×15 mm and was located in the left scapular region (Fig. 1). Thorough clinical examination did not reveal any evidence of tumors located elsewhere or any pertinent past clinical history. No history of significant or hereditary diseases in the family were reported. A punch biopsy was performed by the clinician. Spindle cells with an eosinophilic cytoplasm were observed under high-power examination (Fig. 2). Immunohistochemical studies were performed and the cells stained strongly positive for smooth muscle actin (SMA) (Fig. 3). As a result, the patient was diagnosed with pilar leiomyoma.

This study was approved by the ethics committee of the University of Rize, Turkey. The patient consented to the publication of this study.

## Discussion

The anatomical distribution of cutaneous leiomyomas is extensive. Leiomyomas may present clinically as either solitary or multiple lesions that have a skin-colored or red surface, and are most commonly located in the extremities (7). Solitary and multiple pilar leiomyomas arise from arrector pili muscles (8). Pilar leiomyomas are the most common type of leiomyoma and range from 2 to 20 mm in diameter. When multiple leiomyomas exist, these typically consist of red-brown grouped papules, commonly located on the trunk or the extremities (9). Cutaneous leiomyomas may be asymptomatic, but are typically extremely painful (3). The pain experienced may be spontaneous or as a result of exposure to cold, pressure or emotional stress (3,10). The diagnosis of cutaneous leiomyomas may be accomplished by microscopic examination of a hematoxylin and eosin-stained biopsy of the papule or nodule (10). Tumors in each classification have distinct clinical and/or histologic characteristics (4). Pilar leiomyomas are non-capsulated, circumscribed dermal tumors composed of numerous fascicles of smooth muscle in an interlacing and whorled arrangement (9).

While solitary lesions may be easily treated by surgical excision, multiple lesions covering large areas are more difficult to treat (3). Our patient was referred to a plastic surgeon for surgical treatment.

In conclusion, although cutaneous leiomyoma is a rare disorder we identified a case of pilar leiomyoma in a young female. In the present case, a careful clinical assessment led to the correct diagnosis and therapy. Although cutaneous leiomyomas occur more frequently in adults, we suggest that leiomyoma ought to be considered in the differential diagnosis of any cutaneous or mucosal mass in children.
